# Practices and behaviors of professionals after falls in institutionalized elderly with and without cognitive decline

**DOI:** 10.1590/1980-57642020dn14-010010

**Published:** 2020

**Authors:** Cristina Lavareda Baixinho, Maria dos Anjos Dixe

**Affiliations:** 1 School of Health Sciences, Centre for Innovative Care and Health Technology (ciTechCare), Polytechnic Institute of Leiria (IPLeiria), Leiria, Portugal.; 2 The Health Sciences Research Unit: Nursing, Nursing School of Coimbra, Coimbra, Portugal.; 3 Escola Superior de Saúde, Instituto Politécnico de Leiria, Portugal.

**Keywords:** accidental falls, elderly, caregivers, home for the aged, cognitive dysfunction, psychometrics, acidentes por quedas, idosos, cuidadores, instituição de longa permanência para idosos, disfunção cognitiva, psicometria

## Abstract

Falls are a major problem in nursing homes due to their high prevalence and impact on the functioning of elderly.

**Objective:**

This study aims to: a) construct and validate a scale for assessing the practices and behaviors of professionals from nursing homes after falls in elderly; b) describe practices and behaviors after falls; and c) associate practices and behaviors with professionals’ length of experience, training and age.

**Methods:**

This is a correlational study, conducted in a sample of 152 professionals from six nursing homes. The study adhered to all of the Declaration of Helsinki principles.

**Results:**

The scale constructed has a Cronbach’s alpha of 0.938. The 12 items of the scale are grouped into two factors. The most expressive indicators are the communication of fall episodes that result in severe injuries (4.64 ± 0.812) and the communication of falls that result in injuries and need intervention from health technicians (4.61 ± 0.832). We found no significant statistical difference between length of professional experience, training and age when associated with professional practices and behaviors after falls in elderly (p > 0.05).

**Conclusion:**

Future studies should investigate the association of post-fall professional practices and behaviors with fear of another fall, fall recurrence, and changes in functioning of the elderly following a fall.

## RESUMO

As quedas são um problema grave nas instituições de longa permanência para idosos nos lares devido à elevada prevalência e consequências na funcionalidade dos residentes.

**Objetivo:**

Este estudo tem como objetivos: a) Construir e validar uma escala para avaliar práticas e comportamentos de profissionais das instituições de longa permanência para idosos após a queda; b) Descrever as práticas e comportamentos dos profissionais após a queda e c) Associar as práticas e comportamentos ao tempo de exercício profissional, formação e idade do profissional.

**Métodos:**

Estudo correlacional, realizado com uma amostra de 152 profissionais de seis instituições. Durante este estudo, cumprimos todos os princípios da declaração de Helsínquia.

**Resultados:**

A escala construída possui um alfa de Cronbach de 0,938. Os 12 itens são distribuídos em dois fatores. Os indicadores com maior expressão são a comunicação de episódios de queda, resultando em lesões graves (4,64 ± 0,812) e a comunicação de queda, resultando em lesões e necessitando de intervenção dos técnicos de saúde (4,61 ± 0,832). Não encontramos diferença estatística significativa entre o tempo de experiência profissional, formação e idade, quando associados às práticas e comportamentos profissionais após queda de idosos (p > 0,05).

**Conclusão:**

Estudos futuros devem associar práticas e comportamentos profissionais após uma queda ao medo de uma nova queda, à recorrência e à evolução da funcionalidade do idoso após a queda.

Accidents such as falls are assumed to be a determinant of loss of quality of life in the elderly, due to the resulting injuries, comorbidity, temporary and permanent disabilities, and functional decline. Falls are considered the main cause of restrictions in activities of daily living for this age group, especially among individuals with cognitive decline, for whom it is a significant contributory factor to their dependence.^1^^,^ 2

Although this in itself is cause for concern, the problem is compounded by significant post-fall consequences like fear and post-fall anxiety syndrome, which translate into a loss of self-confidence and the self-imposition of limitations both at home and within institutions.^1^

The relationship between falls and the institutionalized elderly is complex due to its direction – whether falls are the only cause or just one factor in deciding whether to admit an elderly person to a nursing home. A study conducted in the USA concluded that 40.2% of elderly had experienced pre-institutionalization falls,^3^ but that falls should also be seen as a consequence of institutionalization, given that they occur at a higher rate^4^^,^ 5 and lead to more harmful consequences compared to elderly in the community.^1^^,^ 5 Contributing factors to this increase in prevalence include greater dependence, chronic disease, the environment, cognitive decline and the presence of caregivers.^2^^,^ 5 ^,^ 6

An unfamiliar environment and the presence of other people hinders risk identification and control, increasing the risk of a fall, particularly in the first few days of institutionalization, when one in five newly-admitted elderly residents experiences a fall.^3^ It should also be noted that in the first few days of admission, the elderly may feel less confident performing daily living activities, which contributes to increased risk.

Although there is no research focusing directly on practices and behaviors, studies do report unsafe behaviors as a cause of falls, and in this sense, it is worth monitoring this behavior; indeed, in order to improve programs, it is necessary to first improve attitudes and behaviors and understand the organizational characteristics that influence these attitudes and behaviors.^7^

Failure to report is one example of unsafe behavior^8^ that calls for further research because even in the absence of injury, after a fall episode seniors may develop a fear of falling again. This leads to activity limitation, resulting in reduced mobility and physical fitness, which in turn further increases the risk of fall.^6^

The change in epidemiological profile among the elderly – and the associated increase in accidents, mortality, and morbidity – clearly points to the importance of new health models that invest in promoting active aging, the prevention of falls, and a positive cost-benefit relationship, while improving risk identification, the provision of information and assistance to the elderly, and the treatment of after-effects and prevention of post-fall syndrome.^4^^-^ 7 As affirmed by some researchers, it is the responsibility of professionals to decrease event incidence, providing care and striving to ensure the safety and wellbeing of the elderly.^5^^,^ 8 ^-^ 10

With this in mind, the objectives of this study were: a) to construct and validate a scale for assessing the practices and behaviors of professionals in nursing homes after elderly falls (SAPBAEF); b) to describe the practices and behaviors of professionals after falls in institutionalized elderly; and c) to determine the association of practices and behaviors after falls in institutionalized elderly with health professionals’ length of experience, training and age.

## METHODS

The absence of a scale in the literature review ruled out the possibility of administering a pre-existing instrument to assess the variables studied. Therefore, we constructed and validated a scale for practices and behaviors after elderly falls (SAPBAEF).

The construction of this scale followed these steps: definition of the object of assessment, data collection from databases, context observation, interviews with experts, material selection to define items to be included in each scale, creation of a scale, scale assessment by experts, pre-test, reconstruction of scales, application and validation.^11^

A Likert-type scale included 15 initial items, based on evidence and interviews conducted with three expert nurses with more than five years’ professional experience. Respondents rated their practices based on five frequency options (never, rarely, sometimes, often, and always).

The pre-test was conducted in a convenience sample of 23 professionals.

To define the sample size and assess the instrument’s psychometric properties, we opted for at least five respondents per item.^12^ The institutions to be included in the study had to provide care to elderly persons with cognitive decline and the inclusion criteria defined for the caregivers required that they were professionals directly involved in elderly care and provided free consent to participate in the study. We excluded participants that had management or organizational functions and those working exclusively in home care jobs or day centers.

In conducting this study we respected the ethical principles of the Helsinki Protocol; participants provided their informed consent and their privacy and confidentiality were respected. The study was approved by the Research Ethics Committee of the Catholic University of Portugal (UCP).

The scale was distributed by one researcher in six institutions that consented to the study. After filling them out, professionals placed the scale in one box and the consent form in another box. The boxes were left at the nursing homes for 15 days due to rotating work schedules.

The data was processed using SPSS (Statistical Package for the Social Sciences) version 23.

Reliability was tested through internal consistency analysis, determining the Cronbach’s alpha coefficient.^11^^,^ 12

For the factor analysis of principal components, we used Varimax orthogonal rotation and extracted factors with eigenvalues > 1. We used the Cattell graph or scree plot to determine the number of factors to retain. The Kaiser-Meyer-Olkin (KMO) and Bartlett’s tests were used to assess the quality of the correlations between variables and to test the validity of the factor matrix.^11^^,^ 12

Interpretability and statistical criteria were used in the factor analysis of principal components with Varimax rotation.

Once the relationship between variables had been determined, the Kolmogorov-Smirnov test was applied to evaluate their distribution. It was found that the sample had a non-normal distribution. Non-parametric techniques were used to test the relationship between variables studied, for p < 0.05.^11^

This study is part of the project “Management of fall risks in institutions for elderly people” and was approved by the local ethics committee. Participant anonymity and data confidentiality were protected.

## RESULTS

Of the 232 scales distributed, 152 were completed, which translates to a response rate of 65.52%. The sample was made up of 152 female respondents, with a mean age of 47.02 ± 10.3 and who had worked at the institution for 11.9 ± 8.19 years. Only 75% of the sample had been trained in elderly falls before or during their professional careers, and of those, 82.6% learned about post-fall care.

### Reliability

Initially this scale included 15 items; after determining the psychometric characteristics, 12 items were retained, with a Cronbach’s α of 0.938. Item-total correlation without the item itself ranged from 0.607 to 0.877, and the Cronbach’s α coefficient without the item itself ranged from 0.927 to 0.937. These results demonstrate good internal consistency (Table 1).

**Table 1:** Pearson's item-total correlation and Cronbach's α of items, without the item itself, Lisbon, Portugal (n=152).

Number and content of items	Pearson's item-total correlation without the item itself	Cronbach's α without the item itself
1. I try to determine the causes of the fall	.607	.937
2. I try to determine what the person was doing at the time of the fall	.676	.935
3. I increase supervision for the elderly	.752	.932
4. I check if the elderly person is afraid of falling again	.669	.935
5. I record the fall episode in the incident book	.857	.928
6. I report how the fall happened in the incident book	.860	.928
7. I record the time of the fall	.734	.933
8. I describe the injuries resulting from the fall in the incident book	.877	.927
9. I communicate to health technicians falls that did not cause injuries	.665	.936
10. I communicate to health technicians falls resulting in injuries that need little or no care	.649	.936
11. I communicate to health technicians falls resulting in injuries that need intervention	.723	.933
12. I communicate to health technicians falls resulting in fractures and loss of consciousness	.668	.935
Total Cronbach's a	0.938	

### Construct validity

The factor analysis with Varimax rotation determined that the 12 items could be grouped into two factors that explain 69.639% of total variance: 38.856% for the first factor and 30.783% for the second factor (Table 2).

**Table 2:** Analysis of principal components of the SAPBAEF with Varimax rotation and Kaiser normalization, Lisbon, Portugal (n=152).

Number and content of items	H2	F1	F2
1. I try to determine the causes of the fall	.614	.774	
2. I try to determine what the person was doing at the time of the fall	.654	.781	
3. I increase supervision for the elderly	.651	.661	
4. I check if the elderly person is afraid of falling again	.582	.707	
5. I record the fall episode in the incident book	.814	.757	
6. I report how the fall happened in the incident book	.810	.753	
7. I record the time of the fall	.633	.680	
8. I describe the injuries resulting from the fall in the incident book	.836	.753	
9. I communicate to health technicians falls that did not cause injuries	.657		.756
10. I communicate to health technicians falls resulting in injuries that need little or no care	.684		.793
11. I communicate to health technicians falls resulting in injuries that need intervention	.796		.846
12. I communicate to health technicians falls resulting in fractures and loss of consciousness	.626		.707
Kaiser-Meyer-Olkin measure	.900		
Bartlett's test of sphericity	1575.105; p < .001		

The possible scores on the SAPBAEF range from 12 to 60 points.

The first factor relates to professionals’ practices and behaviors after a fall episode and the second describes professionals’ communication of practices and behaviors regarding a fall episode to health technicians of the institution.

### Professionals’ practices and behaviors after falls in elderly 

The assessment scale for practices and behaviors after falls in elderly has two dimensions. In the dimension relating to professionals’ practices and behaviors after a fall episode, the three most highly rated indicators were: trying to determine what caused the fall (4.53 ± 0.769), describing the injuries resulting from the fall in the incident book (4.53 ± 0.951) and recording the fall episode in the incident book (4.54 ± 0.936) (Table 3).

**Table 3:** Characterization of professionals' practices and behaviors after a fall in elderly, Lisbon, Portugal (n=152).

Number and content of items	Mean	SD
1. I try to determine the causes of the fall	4.53	.769
2. I try to determine what the person was doing at the time of the fall	4.45	.828
3. I increase supervision for the elderly	4.50	.824
4. I check if the elderly person is afraid of falling again	4.32	.926
5. I record the fall episode in the incident book	4.54	.936
6. I report how the fall happened in the incident book	4.50	.916
7. I record the time of the fall	4.35	1.111
8. I describe the injuries resulting from the fall in the incident book	4.53	.951
9. I communicate to health technicians falls that did not cause injuries	4.22	1.079
10. I communicate to health technicians falls resulting in injuries that need little or no care	4.32	0.892
11. I communicate to health technicians falls resulting in injuries that need intervention	4.61	0.832
12. I communicate to health technicians falls resulting in fractures and loss of consciousness	4.64	0.812
Total (12-60)	53.51	10.87
Factor 1 - activities performed after a fall episode	35.72	7.26
Factor 2 - communication to health technicians of the institution	17.79	3.61

More expressive indicators were found in the second factor (Table 3). In descending order, these were: communicating fall episodes resulting in severe injuries (4.64 ± 0.812) and communicating falls requiring intervention to health technicians (4.61 ± 0.832). By comparing the mean of each indicator, we found a higher probability of reporting a fall that resulted in serious lesions like fractures and loss of consciousness (4.64 ± 0.812) than a fall resulting in no injuries (4.22 ± 1.079).

We found no statistically significant difference between professionals’ job experience, training and age, and their practices and behaviors after falls in elderly (p > 0.05).

## DISCUSSION

The validated scale constructed to measure the latent variable in the study – nursing home professionals’ practices and behaviors after a fall episode – showed very good internal consistency (Cronbach’s α 0.938). The 12 items are distributed in two factors, the first enables an assessment of behavior after a fall episode and the second assesses incident communication to health technicians in the institution.

After a fall episode, even when there is no injury, seniors may develop a fear of falling again. This can lead to activity limitation, which in turn reduces mobility and physical fitness, further increasing the risk of falls.^8^ Out of fear of recurrent falls, caregivers tend to limit elderly activity, especially in those with dementia.^1^^,^ 7 ^,^ 13

A study that investigated the association between subtype and severity of dementia and falls, and the role of poor gait in falls across the spectrum and the subtype of dementia, concluded that non-Alzheimer disease (AD) dementia patients were twice as likely to have had a previous fall than healthy older adults, whereas patients with AD had a similar rate of previous falls to healthy older adults.^13^ Each group of individuals with cognitive decline, regardless of the severity of decline, had an increased odds ratio (OR) for falls in comparison with cognitively healthy individuals.^13^

Comparing the total obtained for the sample to the highest possible score on the scale (53.51 out of 60 points) allows us to affirm that, while professionals’ post-fall practices and behaviors are positive, they are not always maintained.

Factor 1 scored 35.72 points out of 40, indicating concern among health teams to understand what caused the falls and which activities elderly residents were performing at the time, and to assess the fear of another fall, record what happened and describe resulting injuries in the incident book.

A vital aspect of fall prevention is communicating and reporting a fall episode to prevent recurrence.^1^^,^ 4 The indicator “record the fall episode in the incident book” (4.54 ± 0.936) contrasts with another study, also conducted in Portugal, where researchers, upon reviewing the written records of a nursing home, concluded that there was no data on post-fall measures in 81.3% of situations,^1^ suggesting lack of importance concerning the communication of fall episodes.^1^ Future studies should explore what influences professionals’ post-fall practices, behaviors, and communication with health technicians from the institution.

Documentation is an important measure in managing fall risk. A previous study that assessed a fall prevention program in a hospital environment found that using documentation systems like exclusive care plans and registries for accidents can be considered prevention measures^14^ because they focus professionals’ attention, raise awareness about the existence of fall risk factors and promote actions to eliminate or minimize falls. International guidelines recommend that professionals from nursing homes perform a complete assessment for each incident, with documentation and reports on all associated aspects, in accordance with institutional protocol or guidelines on the immediate post-fall process.^1^^,^ 14

Other studies also discuss the possibility of underreporting fall episodes, believing this to be less prevalent in cases with severe injuries.^15^ Elderly self-stigmatization can influence self-reporting of falls by introducing false positives and false negatives, meaning that not all episodes are reported.

In addition, we found that professionals increase elderly supervision following a fall. Future studies should explore the association between this increase in supervision and the decrease in elderly activity and even physical restriction of mobility, particularly in elderly with dementia.

Physical restriction of mobility is a concern in elderly institutions and is often used as a fall prevention measure. Cognitive impairment, severe mobility problems and a greatly restricted capacity to perform daily living activities are determining factors in the decision to implement restraint systems.^2^^,^ 7

Each nursing home has specific needs determined by elderly characteristics, number of residents, knowledge and experience, prevention practice, fall recording and reporting, and the location and architectural characteristics of buildings,^4^ which can influence post-fall practices and behaviors.

The lowest scoring indicator in the first factor is checking whether the elderly person is afraid of falling again (4.32 ± .926). Despite fear of falling being frequent in nursing homes,^16^^,^ 17 interventions to decrease fear are rare.^18^ A study examining the efficacy of cognitive-behavioral strategies with or without exercise to reduce fear of falling in elderly residents of nursing homes suggested that interventions (education, helping to understand that fall risk and fear can be controlled, defining objectives to increase activity and eliminating environmental barriers) helped the elderly to manage their fear of falls, decrease their depressive inclination, and improve their mobility and physical strength.^17^

The second scale factor obtained a score of 17.76 out of 20, which indicates good practices in communicating fall episodes to health professionals. Communication is a central element in fall prevention programs.^19^ Communication between professionals – but also between residents and professionals – about preventive measures and health promotion can ensure that appropriate and specific interventions are carried out to decrease the incidence of falls.^10^^,^ 19

Previous work has shown that team training can help reduce falls^19^ and the outcomes of this study lend further weight to the need for investment in training of teams, including post-fall reporting and action.^20^

Client safety in nursing care depends on the high quality of care provided by nurses and other professionals, and excellent communication and team work. As a result, communication between professionals is an ethical imperative in providing and managing care.

This study has some limitations associated with the study population and data collection instrument. Institutions and professionals were selected purposively, which may affect data generalization. The instrument was self-administered with a Likert scale, which increases the risk of the responses tending towards the socially desirable.

Despite these limitations, this study presents a scale with excellent internal consistency (Cronbach’s α 0.938) that can be used to evaluate nursing home professionals’ practices and behaviors after falls in elderly. Future studies should explore the association of total scores on the scale and each dimension with the prevalence of recurrent falls.
